# The Impact of the COVID-19 Pandemic on Young Adults with Autism Spectrum Disorder: A Systematic Review

**DOI:** 10.3390/healthcare13111216

**Published:** 2025-05-22

**Authors:** Azahara Leonor Miranda Gálvez, Antonia Pilar Pacheco-Unguetti

**Affiliations:** 1 Department of Psychology, University Loyola, 14004 Córdoba, Spain; 2 Department of Personality, Assessment and Psychological Treatment, Mind, Brain and Behavior Research Center (CIMCYC), University of Granada, 18071 Granada, Spain; tpacheco@ugr.es

**Keywords:** autism spectrum disorder, COVID-19, young adults, mental health, socialization, employment, autonomy, digital interventions

## Abstract

Background/Objectives: The COVID-19 pandemic and related public health measures significantly disrupted daily life, with profound consequences for individuals with Autism Spectrum Disorder (ASD). Young adults with ASD faced unique challenges due to disruptions in routines, employment instability, limited access to essential services, and increased social isolation. While some individuals benefited from reduced social pressures and the adoption of remote work, many experienced heightened anxiety, behavioral difficulties, and declines in autonomy. This systematic review examines the impact of the pandemic on young adults with ASD, focusing on key domains such as autonomy, employment, service accessibility, socialization, emotional regulation, and overall well-being. Methods: This review followed the PRISMA 2020 guidelines, and its protocol was pre-registered in the PROSPERO database. A search was conducted in four databases—PubMed, Scous, Web of Science, and PsycInfo—as well as in specialized journals in the field. Results: Eight studies met the inclusion criteria and were included in the final synthesis. The findings highlight significant disruptions in daily life, increased dependence on caregivers, and difficulties in maintaining structured activities. However, technology-assisted interventions, including virtual therapies and remote work opportunities, played a role in mitigating some adverse effects. Conclusions: Despite the heterogeneity in methodologies, this review underscores the urgent need for targeted interventions to support young adults with ASD during crises. Future research should focus on long-term consequences and developing inclusive policies that enhance resilience, access to services, and social integration.

## 1. Introduction

### 1.1. Background of the COVID-19 Pandemic

The COVID-19 pandemic, caused by coronavirus (SARS-CoV-2), was officially declared a global health emergency by the World Health Organization (WHO) on 11 March 2020 [1]. By that date, global data on COVID-19 was limited and constantly evolving. However, more than 118,000 confirmed cases and 4291 deaths had been reported in 114 countries [2]. Since then, according to the most recent data from the WHO, by 24 December 2024, over 776.8 million confirmed cases and more than 7 million deaths from COVID-19 had been recorded worldwide [3]. It has been considered the greatest global challenge since World War II.

Following the declaration, a wide range of public health measures were swiftly introduced to combat the spread of the virus and reduce its impact on global health systems. These measures, recommended by health authorities, included isolation protocols, quarantine for those exposed or symptomatic, social distancing to limit close contact between individuals, and community containment strategies to prevent further outbreaks [4]. These interventions were essential in controlling the spread of the virus, protecting vulnerable populations, and minimizing the strain on healthcare systems worldwide [5]. Although these measures were essential for protecting physical health, they also triggered significant shifts in daily routines, social interactions and emotional well-being, with notable psychological consequences.

### 1.2. Autism Spectrum Disorder: Historical Context, Diagnosis, and Core Features

Autism was first described by Leo Kanner [6] in 1943, identifying key features like social withdrawal and communication difficulties. Around the same time, Hans Asperger [7] independently described a similar condition, later known as Asperger’s syndrome. Before the Diagnostic and Statistical Manual of Mental Disorders 5th Edition (DSM-5) [8], Autism Spectrum Disorders (ASD) were classified as separate conditions within Pervasive Developmental Disorders (PDD), causing inconsistencies. The DSM-5 unified these conditions under ASD, reorganized the diagnostic criteria into two core domains, and introduced three severity levels based on required support [8]. Consequently, at present, ASD is a heterogeneous neurodevelopmental disorder, characterized by a broad range of symptoms that vary significantly across individuals [9,10]. Core features include communication deficits, such as difficulties with social reciprocity, nonverbal communication (e.g., understanding gestures, eye contact, and body language), and forming relationships. Additionally, individuals with ASD often display restrictive and repetitive behaviors, such as stereotyped movements, resistance to change, and altered sensory sensitivities [11].

This variability in symptoms, both in severity and impact on daily functioning, underscores the critical importance of routines and structure for individuals with ASD [12]. Stability and predictability are fundamental to their well-being and ability to navigate social and familial environments [13,14]. For many, maintaining consistent routines plays a key role in managing anxiety and adapting to changes [15].

Moreover, individuals with ASD frequently experience high rates of co-occurring physical and mental health conditions, such as anxiety and depression, which contribute to the complexity of their needs [16,17].

### 1.3. Impact of COVID-19 on Individuals with Autism Spectrum Disorder (ASD)

The COVID-19 pandemic has had a significant impact on mental health worldwide [18]. Various studies have reported increased levels of anxiety, loneliness, and psychological distress, along with a decline in life satisfaction and overall well-being [19,20,21,22]. This effect has been particularly severe in vulnerable populations facing heightened challenges due to preexisting conditions [23,24]. Among these populations, individuals with autism spectrum disorder (ASD) experienced notable disruptions in their daily lives, primarily due to health-related restrictions that interfered with their established routines. For children with ASD, changes in school and therapy schedules led to increased stress, as their firm reliance on predictable structures made adaptation more difficult [25,26,27].

The COVID-19 pandemic also disrupted several aspects of the person–environment fit, such as routine stability, family dynamics, and learning or working conditions—all of which are essential for the well-being of individuals with ASD [28]. Since adherence to routines is a core characteristic of autism, sudden changes have been associated with increased anxiety and depression [29]. In addition to fears of contracting the virus, containment measures imposed further psychological burdens, particularly for those with ASD, who often find unexpected transitions especially challenging [30,31,32,33,34].

Younger children were particularly affected, as disruptions to their familiar patterns further exacerbated these difficulties [8,35,36]. Families reported that while dinnertime routines became more chaotic, structured bedtime routines provided some positive interactions and rituals [35]. Additionally, disruptions to daily routines were also linked to increased comorbid symptoms in children with ASD, with studies showing a correlation between routine stability and both internalizing (e.g., anxiety, withdrawal, crying) and externalizing (e.g., aggression, noncompliance, failing to finish tasks, argumentativeness) behaviors [36].

In contrast, some autistic adults experienced a reduction in social stress, as the decrease in mandatory social interactions during the pandemic proved beneficial [37]. It has been suggested that ASD is associated with an impaired stress response mechanism, particularly in socially stressful situations [38,39]. While this atypical stress response appears to be context dependent [40], it remains unclear whether individuals with ASD exhibit distinct emotional responses to pathogen-related stressors, such as those triggered by the COVID-19 pandemic.

The vulnerability of individuals with ASD during the pandemic has been considerable, leading to behavioral challenges and psychological distress [25,41,42]. Several studies have examined the impact of pandemic restrictions on individuals with ASD, focusing on key aspects of their daily lives, such as changes in routines, behavior, emotions, socialization, and autonomy. These studies have also highlighted the effects of disrupted access to support services, education, and healthcare, which further compounded the challenges faced by individuals with ASD during the pandemic. The findings suggest that these disruptions have led to increased behavioral issues, heightened stress levels, and a decline in social interactions, significantly affecting overall well-being [37,43,44,45,46].

### 1.4. Research Gap and Study Aims

Although the general impact of the COVID-19 pandemic on individuals with ASD has been extensively studied, there is limited research specifically focusing on young adults. This is an important gap, since adulthood is a period of significant transition, marked by increased demands in multiple areas such as employment, independent living and social integration [47,48]. Additionally, the pandemic introduced additional challenges for this age group that require more in-depth study.

While several reviews have explored the impact of COVID-19 on children [49,50,51] and adolescents with ASD [52,53], less is known about its impact on young adults, who face unique challenges related to independence, employment, and social relationships. Addressing this gap is crucial for developing effective support systems and interventions tailored to the needs of young adults with ASD in post-pandemic recovery. Therefore, the proposed systematic review had the following main aim: to explore the impact of the COVID-19 pandemic on young adults with ASD. To achieve this, we formulated two specific research questions:

(1) What specific effects did the COVID-19 pandemic have on young adults with ASD?
(2) How did these effects evolve throughout the pandemic in this population, considering immediate impacts as well as short-, medium-, and long-term changes?

## 2. Materials and Methods

This systematic review (SR) was carried out in line with the methodologies outlined in the Cochrane Handbook for Systematic Reviews to ensure rigorous and standardized procedures throughout the review process (https://training.cochrane.org/handbook, accessed on 6 March 2025). The findings were reported following the Preferred Reporting Items for Systematic Reviews and Meta-Analyses (PRISMA) guidelines [54], to maintain transparency and consistency in the presentation of the review results. Additionally, the review protocol was registered in the PROSPERO database on 7 March 2025, with the registration number CRD420250647804, ensuring compliance with the established protocol.

### 2.1. Search Strategy

Eligible articles were initially selected from four online databases: PubMed, Scopus, Web of Science, and PsycInfo on 27 March 2025 (The original protocol defined young adults as ages 18–25. However, due to substantial variability in the age ranges used across studies, we removed this restriction to ensure a more inclusive and representative synthesis. This update was registered in PROSPERO), based on predefined inclusion and exclusion criteria. To ensure a comprehensive review, the search was broadened to include additional articles from specialized autism journals, such as Autism Research, Journal of Autism and Developmental Disorders, and Autism in Adulthood.

The following PECO framework was implemented: (1) Patient: young adults diagnosed with Autism Spectrum Disorder (ASD) according to the criteria outlined in the DSM-5; (2) Exposure: the exposure of interest is the COVID-19 pandemic (effects of lockdowns, social distancing, and disruptions to daily routines); (3) Comparison: none; (4) Outcome: changes in symptoms in individuals with ASD (including difficulties or improvements in social communication and interaction, increase in restricted interests and repetitive behaviors); psychological impact (changes in mental health, mood states, behavior and overall well-being). These outcomes were based on the domains reported in the DSM-5 [8].

The inclusion criteria for this study are empirical quantitative and/or qualitative research examining the impact of the COVID-19 pandemic and its associated restrictions on mental health or well-being, based on self-report and/or observation/proxy-report, up to the present. The paper must explicitly mention young adult(s) in the “Method” section, and the sample should consist of young adult(s). Studies published in English or Spanish from any country are eligible, provided they are published in a peer-reviewed journal or as a pre-print on a pre-print service provider. In terms of exclusion criteria, studies focusing exclusively on adults, children, or individuals with other diagnoses, such as intellectual disability, will be excluded unless they meet specific conditions. Specifically, a study will be considered for inclusion if it provides a distinct and separate analysis of data pertaining to autistic adults or if autistic adults represent at least 50% of the total sample. Furthermore, studies that examine variables related to autism but not aligned with the objectives of this review (e.g., medical issues) will be excluded, as well as articles that do not provide full-text access.

The search strategy was further refined using the following filters: language (English and Spanish), publication date (since December 2019 (although the World Health Organization (WHO) officially declared the COVID-19 pandemic on 11 March 2020, the initial outbreaks of the SARS-CoV-2 virus emerged in December 2019. By extending the review period from this initial date to the present, the aim is to capture the complete evolution of the pandemic’s impact on young adults with autism. This is the reason the search includes from 2019 to present literature)) and the population being young adults. In the databases, we identified relevant studies using the following key words: “autism”, “autism spectrum disorder” (ASD), “autistic young adults”, “COVID-19”, “SARS-CoV-2”, “coronavirus”, “young adult”, and “emerging adults”. These terms were specifically included in the title and abstract fields. The databases were queried as follows: Pubmed: (autism [TIAB] OR “autism spectrum disorder” [TIAB] OR ASD[TIAB] OR “autistic young adults” [TIAB]) AND (“COVID-19” [TIAB] OR SARS-CoV-2[TIAB] OR COVID [TIAB] OR coronavirus [TIAB]) AND (“young adult”[TIAB] OR “young adults” [TIAB] OR “emerging adults” [TIAB]); Scopus: TITLE-ABS-KEY(autism OR “autism spectrum disorder” OR ASD OR “autistic young adults”) AND TITLE-ABS-KEY (“COVID-19” OR SARS-CoV-2 OR COVID OR coronavirus) AND TITLE-ABS-KEY (“young adult” OR “young adults” OR “emerging adults”); Web of Science: TS = (autism OR “autism spectrum disorder” OR ASD OR “autistic young adults”) AND TS = (“COVID-19” OR SARS-CoV-2 OR COVID OR coronavirus) AND TS = (“young adult” OR “young adults” OR “emerging adults”) and PsycInfo: TIAB (autism OR “autism spectrum disorder” OR ASD OR “autistic young adults”) AND TIAB (“COVID-19” OR SARS-CoV-2 OR COVID OR coronavirus) AND TIAB (“young adult” OR “young adults” OR “emerging adults”).

In the specialized autism journals, the search was performed utilizing the following keywords: “young adults” and “COVID-19,” with a focus on identifying relevant articles that included these terms specifically in the title and abstract.

### 2.2. Screening and Eligibility

One author (ALM) conducted the initial research and identified 164 records from databases and 22 from specialized autism journals. These records were imported into an Excel spreadsheet for organization. Duplicate entries were removed after verifying titles and authors, resulting in 157 unique records for screening.

In the second step, ALM reviewed titles and abstracts, excluding 100 records that did not align with the inclusion criteria. In the next stage, the full-text articles of the remaining 57 potentially relevant studies were assessed independently by both (ALM and APP-U) to determine eligibility. Following this thorough eligibility assessment, 49 studies were excluded. Finally, the 8 studies selected for inclusion in this SR underwent data extraction performed by ALM using a structured Excel sheet. APP-U then cross-checked the extracted data to ensure accuracy. ALM systematically extracted data using a custom-designed tool to capture key study characteristics (e.g., authors, publication year, journal name, DOI, title, objective, study design) and sample details (e.g., sample size, gender distribution, age range, country of origin and reported outcomes).

### 2.3. Methodological Quality Assessment

The risk of bias in the included studies was independently assessed by two reviewers (ALM and APP-U) using the Critical Appraisal Skills Programme (CASP) Checklists [55]. The appropriate checklist was selected based on each study’s design to evaluate methodological quality, with the aim of minimizing risk of bias and ensuring the credibility and generalizability of the results obtained. This validated tool, widely used in systematic reviews, provides a structured framework for assessing key methodological aspects, including research objectives, literature review depth, study design justification, analytical approach, efforts to minimize bias, and identification of confounding variables [56,57,58].

### 2.4. Variables Studied

The variables examined in this SR reflect key aspects of life that are particularly relevant for individuals with ASD. To facilitate analysis, the findings are organized into three main areas: Daily Life and Accessibility, Social and Emotional Impact, and Behavioral and Cognitive Outcomes. This structure highlights how the pandemic influenced daily functioning, emotional well-being, and behavioral responses in this population. Regarding missing or unclear information, studies that did not provide sufficient details on key outcomes (such as participant characteristics, the specific impact of the pandemic on daily life, or relevant behavioral measures) were excluded from this review. Only studies that clearly defined and reported on these variables were included in the final analysis, ensuring the comparability of the evidence. Studies that did not provide sufficient details on key outcomes (i.e., participants characteristics, specific impact of the pandemic on daily life), will be excluded from this SR.

## 3. Results

**Figure 1:**
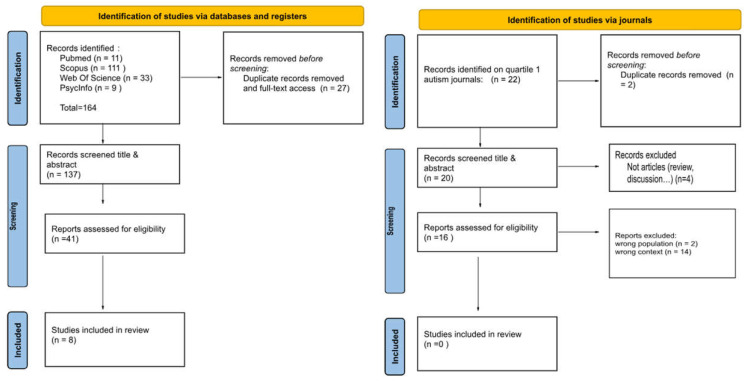
PRISMA flow chart outlining the steps involved in the development of this SR.

A total of 137 studies were initially identified from the four databases. An updated search, conducted 2 weeks later, included additional quartile 1 autism journals, which resulted in the identification of 22 more papers, bringing the total of 156 studies. However, after screening all the studies and applying the inclusion criteria, only eight papers were included in this SR. Studies focusing exclusively on autistic traits or undiagnosed individuals were also excluded. The PRISMA flow chart outlining the steps involved in the development of this SR is presented below (Figure 1).

### 3.1. Characteristics of Studies

General information concerning the eligible studies is summarized in Table 1. The studies included in this review varied in terms of sample size, age range of participants, and methodological approaches. All eight studies included were published between 2020 and 2024. Six (75%) were longitudinal and two (25%) were cross-sectional studies. A total of 324 participants were analyzed, of whom 185 (57.1%) were male. Among them, 288 were autistic individuals, consisting of 167 males (58.0%), 119 females (41.3%), 1 participant who identified as “other genders” (0.3%), and 1 as non-binary (0.3%).

All the studies involved comparisons of different groups: four (50%) compared before and during the COVID-19 restrictions, one (12.5%) compared autistic individuals with and without co-occurring mental health conditions during COVID-19, one (12.5%) compared ASD participants with control groups, one (12.5%) examined ASD individuals with employment changes during the pandemic versus those without, and one (12.5%) compared individuals with ASD who had access to in-person care versus those who only received telehealth.

Regarding the period of the COVID-19 pandemic examined, all included studies focused on specific phases of the pandemic. For instance, Brondino et al. [59] analyzed the initial stage (19 February–4 March 2020), Davidson and Pfeiffer [60] addressed the early phase (summer 2020), and Mosquera et al. [61] examined the first lockdown. Pfeiffer et al. [62] distinguished two periods: 20 March–2 April and 20 April–3 May. Schnitzler et al. [63] assessed a separate phase, while Taylor et al. [64] focused on May 2020. Tovin and Núñez-Gaunaurd [65] studied the quarantine lockdown, and Valenti et al. [66] compared three time points: during lockdown (T0), 6 months later (T1), and after 1 year (T2). This methodological heterogeneity complicates synthesis and calls for more detailed consideration in the results and discussion.

### 3.2. Daily Life and Accessibility

Five studies examined the daily routines and accessibility challenges faced by autistic individuals during the COVID-19 pandemic [60,61,62,64,66]. These studies highlighted positive and negative aspects of pandemic-induced changes, including shifts in daily activities, employment, personal autonomy, and access to essential services. See Table 2 for a summary of key findings from the reviewed studies.

Changes in daily routines were evident in several studies. For example, Mosquera et al. [61] found that some autistic individuals appreciated the additional time for personal interests and family interactions during the pandemic. Additionally, the social recognition of healthcare workers, such as public applause, provided emotional relief. However, other studies documented significant disruptions in daily activities. Pfeiffer et al. [62] observed an overall reduction in the number of activities, with some individuals engaging only in essential tasks, while others redirected their focus to non-essential activities. These findings indicate that the pandemic altered how individuals structured their time and prioritized their routines, though this trend was not consistent across all participants. Valenti et al. [66] further reported a statistically significant decline in domestic skills, particularly in young adults with autism, who became increasingly reliant on their parents for household tasks. This decline was especially noticeable between the first lockdown and one-year post lockdown and between six months and one year after restrictions eased. While these findings suggest a tendency toward reduced autonomy, the extent and significance of this impact varied by individual and study, highlighting the diverse experiences of this population.

Employment and remote work were also analyzed in two studies specifically addressing employment challenges for young adults with autism during the pandemic [61,64]. A notable shift toward remote work was seen as a positive development, offering increased workplace inclusion opportunities. Remote work provided a more accommodating environment, making employment more accessible for individuals with ASD. Despite this positive shift, employment instability remained a significant issue. For example, Taylor et al. [64] reported that 37.5% of participants experienced employment changes during the first two months of the pandemic. These included reduced work hours (37%), temporary job loss (31.5%), and permanent job loss (18.5%). Additionally, 26.4% of participants faced salary reductions or job loss, while 70.4% encountered either job loss or work-hour reductions. On the other hand, 30% reported new job opportunities, role changes, increased hours, or changes in workplace support, demonstrating a mixed impact on employment outcomes. These findings suggest a tendency toward employment instability during the pandemic; however not all changes were negative, as there were also potential opportunities associated with the shift to remote work. Overall, these results demonstrate the complexity and variability of employment-related experiences for individuals with ASD during this period.

Accessibility of services and transportation was another key concern. Among the reviewed studies, Mosquera et al. [61] was the only one to address the shortage of essential services for individuals with autism. The study highlighted significant difficulties in accessing medical care and social support, which exacerbated existing challenges in healthcare and overall well-being. Transportation was also a significant accessibility barrier. Davidson and Pfeiffer [60] found that transportation issues affected 32% of participants, with no instances in which transportation acted as a facilitator. Pfeiffer et al. [62] reported a significant decrease in travel frequency post-COVID-19. Before the pandemic, individuals used various transportation methods, including public transit, taxis, Uber, and Lyft. However, after the pandemic, most relied solely on walking or driving. GPS tracking data further indicated that mobility levels remained consistently lower during post-pandemic periods compared to pre-pandemic trends, suggesting a tendency toward reduced mobility rather than a definitive decline.

Additionally, the pandemic was linked to a decline in personal autonomy, particularly in self-care behaviors. Valenti et al. [66] found a statistically significant decline in personal hygiene, dressing, and eating habits. The most significant reductions occurred between the first lockdown and six months post lockdown and between the first lockdown and one year later. However, while these findings suggest a general decline, the impact appeared to vary, with younger individuals being more affected. This points to a trend rather than a definitive conclusion, indicating that age may have played a role in the extent of vulnerability to the restrictive conditions of the pandemic.

### 3.3. Social and Emotional Impact

The COVID-19 pandemic had a variable impact on individuals with autism spectrum disorder (ASD), affecting their emotional recognition, social interactions, employment stability, and overall well-being. One key aspect of this impact was emotion perception, as analyzed by Schnitzler et al. [63]. Their findings indicate that while autistic and non-autistic individuals exhibited similar accuracy in identifying emotions in uncovered faces (65% success rate), emotions such as pride, embarrassment, anger, and fear were more challenging to recognize. The use of face masks further reduced accuracy in the ASD group (59.1% ± 7.7), with the addition of sunglasses exacerbating these difficulties. Nevertheless, fear remained the most accurately recognized emotion in both groups.

These findings align with previous research indicating that face masks hinder facial expression recognition, reduce confidence in emotion interpretation, and decrease the perceived intensity of all expressions, with a more significant impact on individuals with high autistic traits (AQ-10) [67]. By obscuring key facial cues, masks increased the risk of emotion misinterpretation, such as confusing disgust with anger or perceiving emotions as neutral. This was particularly challenging for individuals with ASD, who tend to focus more on the mouth than the eyes [68,69], making facial expressions harder to interpret.

The pandemic also intensified social challenges. Restrictions on in-person interactions and increased reliance on nonverbal cues further complicated communication for individuals with ASD, contributing to uncertainty and social isolation [60,66]. This increased dependence on nonverbal cues was largely driven by the widespread use of face masks and the shift to virtual communication. Face masks obstructed access to facial expressions and lip-reading, which are essential for many individuals with ASD in interpreting emotions and social intent. Moreover, autistic traits are associated with lower tolerance for uncertainty and unexpected changes [70,71], making it particularly difficult for individuals with ASD to adapt to measures such as social isolation and routine modifications, thereby increasing stress levels and emotional dysregulation [72].

The social impact of the pandemic was complex: while 47% of individuals with ASD experienced increased discrimination and isolation, 21% reported relief from reduced social pressures [60]. However, for the majority, maintaining relationships remained challenging, exacerbating stress and social anxiety [66]. Longitudinal studies confirmed a significant decline in social interactions from the initial lockdown to one year later.

Although some studies highlight the increase in remote work as an opportunity for workplace inclusion [61], employment stability also emerged as a critical factor influencing mental health. Job loss or reduced working hours were strongly associated with increased depressive symptoms, particularly among those who perceived these changes negatively [63]. Given the well-documented difficulties individuals with ASD face in adapting to change, sudden shifts in employment status have likely contributed to significant psychological distress.

Despite these challenges, technological solutions helped mitigate some adverse effects. Digital platforms facilitated access to essential services and alleviated social isolation [65]. Technology-based interventions also promoted physical activity, with devices such as Fitbit supporting adherence to exercise programs: 80% of participants attended weekly telehealth sessions and achieved their self-identified physical activity goals. While these findings suggest that technology-driven interventions can be valuable resources for maintaining routines and supporting mental health, it is important to note that not all studies reported significant improvements, and the success of such interventions may vary based on individual circumstances and the level of support available. These trends highlight the potential of digital solutions, though further research is needed to confirm their broader applicability and effectiveness in this population.

### 3.4. Behavioral and Cognitive Outcomes

The studies examined various aspects of behavior in autistic individuals, particularly in the context of the pandemic. Despite significant disruptions such as lockdowns, changes in daily routines, and limited access to support services, findings indicated that behaviors related to irritability, social withdrawal, stereotyped actions, hyperactivity, and inappropriate speech remained relatively stable. No significant increases or worsening of these behaviors were observed, suggesting that, while the pandemic introduced multiple stressors, it did not lead to a widespread deterioration in these specific behavioral domains among autistic individuals [59].

Regarding social and cognitive behavior, gaze patterns in autistic and non-autistic individuals showed no significant differences when observing faces for emotion recognition. This suggests that both groups allocated their visual attention similarly, implying that discrepancies in emotional recognition accuracy were not attributable to differences in eye contact or gaze fixation.

Similarly, no notable differences were found in spatial distance regulation between autistic and non-autistic participants, indicating that both groups comparably managed interpersonal space. These results challenge common assumptions regarding social attention and personal space regulation in autism, suggesting that autistic individuals’ behaviors in these specific areas align closely with those of non-autistic individuals [63].

### 3.5. Impact of COVID-19 on Young Adults: Before, During and After the Pandemic

The COVID-19 pandemic had a variable and, in some domains, notable impact on young adults with autism, leading to a progressive decline in autonomy, social interactions, and daily routines across different phases—before, during, and after lockdowns. Among the existing research, only Valenti et al. [66] examined the effects across all three time points, providing valuable longitudinal insight into the evolving effects of the pandemic on this population. Before the pandemic, these individuals maintained stable levels of independence, managing daily tasks such as hygiene, dressing, and eating while engaging in social interactions through structured environments like school, work, or therapy. The onset of the first lockdown, however, was associated with a significant reduction in autonomy and social engagement, as disruptions to daily routines and reduced access to external support increased reliance on caregivers. While some individuals adapted to digital forms of communication, overall social interaction generally declined due to the suspension of in-person activities.

Even a year after the initial lockdown et al. [66] reported that autonomy continued to decline, particularly among younger individuals reliant on structured environments that had not fully resumed. Social relationships remained strained, household routines had not recovered, and domestic skills were significantly lower than pre-pandemic levels. These findings suggest potential long-term impacts of pandemic-related disruptions on daily functioning. They underscore the importance of sustained support to promote the recovery of independence, social engagement, and routine stability. However, further longitudinal research is necessary to better understand the enduring effects of the pandemic and to evaluate the long-term effectiveness of post-pandemic interventions.

### 3.6. Methodological Quality of the Studies and Risk of Bias

Using the Critical Appraisal Skills Programme (CASP) criteria, the methodological quality of the included studies was independently assessed by both authors (see individual evaluations in Supplementary S1). Discrepancies were resolved through consensus. Each study was classified as high (≥70%), moderate (40–69%), or low quality (<40%), based on methodological rigor and risk of bias.

Three studies were rated as moderate quality [59,61,62], reflecting certain methodological limitations that may affect the robustness of their findings. Two studies were categorized as moderate to high quality [60,64], showing solid methodological foundations with minimal risk of bias. Lastly, three studies were deemed high quality [63,65,66], characterized by rigorous design, low risk of bias, and transparent reporting, thus offering the most reliable evidence in this review. However, the evidence had limitations. Sample sizes are often small, which reduces statistical power and limits the generalizability of findings. Additionally, some relevant variables such as comorbidities or level of autism have not been considered. Studies with follow-up are few [64,66], and those that do include follow-up tend to cover short periods.

## 4. Discussion

The COVID-19 pandemic significantly impacted young adults with ASD, affecting their daily routines, social interactions, employment, and emotional well-being. The SR highlights the multiple challenges and adaptations that emerged throughout different pandemic phases, demonstrating the evolving nature of ASD-related symptoms over time. However, when comparing these findings with data from other studies, nuanced patterns and, in some cases, divergent perspectives emerge, enriching the understanding of the pandemic’s impact on this population. The heterogeneity in methodologies, inherent to the autistic population and the transitional nature of young adulthood, was not treated as a limitation but as a defining feature of the current evidence base. To address this, we employed a narrative synthesis approach, allowing for a systematic comparison across studies, identifying shared patterns, and contextualizing divergent findings.

One of the primary issues identified in the systematic review was the disruption of daily routines and accessibility to essential services, resulting in increased dependence on caregivers [63,66]. This aligns with findings indicating that 78.1% of families experienced difficulties managing leisure time, while 75.7% struggled with structured activities [73]. Additionally, parents of children with ASD reported increased psychological distress, often associated with the disruption of services and reduced support during the pandemic [74]. Some studies suggest that disruptions in routine and heightened uncertainty may have affected the expression of ASD-related symptoms and contributed to greater caregiver burden, with possible effects on family dynamics [75]. Furthermore, increased dependency on external support and difficulties with self-care were common challenges faced by individuals with ASD during the pandemic [62,66]. These trends require cautious interpretation, as they may reflect temporary and structural shifts. They also emphasize the need for further research to understand better the extent and persistence of the pandemic’s impact. To address these challenges, the WHO has developed international guidelines to ensure that individuals with disabilities, including those with ASD, are included in health responses to the crisis [2]. Public health policies must address both immediate needs and long-term strategies for the inclusion of people with disabilities in pandemic response and recovery. Additionally, future guidelines should specifically target ASD, considering age-related needs to ensure appropriate and tailored services for individuals at different developmental stages.

However, some studies suggest that the pandemic also fostered certain positive aspects of autonomy in individuals with ASD. Improvements in executive functions among adults with ASD following the isolation period suggest cognitive adaptation to the new reality [76]. Additionally, adopting remote work and virtual health services enhanced stability and structure to their routines, helping some individuals maintain a sense of autonomy [61]. This suggests that while the pandemic introduced significant challenges for individuals with autism, specific individuals were able to adapt and demonstrate resilience, particularly in areas where new systems and support structures were implemented.

The role of caregivers supporting individuals with autism—from childhood through adolescence and into adulthood—must not be overlooked. In the case of children, studies have shown that parents of children with ASD often experience heightened levels of stress, anxiety, fatigue, and depression, mainly due to the demands of caregiving, difficulties accessing adequate support systems, and the persistence of societal biases [77,78,79]. This elevated stress affects caregivers’ health and well-being and compromises their ability to provide optimal care. These challenges place caregivers at higher risk for psychological, behavioral, and physical health concerns, emphasizing the urgent need for tailored support services. Recognizing the prevalence and profound impact of ASD on families is essential to address their needs better. Similar difficulties are encountered by parents and caregivers of autistic adults, highlighting the importance of structured support systems throughout the entire lifespan [80].

Employment instability affected many individuals with ASD, although some reported benefits from remote work [64]. Employment disruptions were particularly detrimental to this population, limiting access to job opportunities and impacting financial well-being [81]. Job loss and financial difficulties were, nevertheless, frequently associated with increased anxiety and stress for both individuals with ASD and their caregivers, potentially influencing family quality of life [75]. These findings underscore the need to design more flexible and inclusive labor policies that enable individuals with ASD to access sustainable employment opportunities in in-person and remote settings. The impact of the pandemic on social interactions and its subsequent effects on anxiety and distress levels were notably ambiguous both within the reviewed studies and in comparison to other existing literature. Some studies indicated an increase in anxiety and social discomfort due to disruptions in daily routines and social isolation [82,83], while others reported a reduction in stress [59,60]. This inconsistency highlights the complex and individualized nature of the pandemic’s impact on mental health, reflecting how social dynamics, coping mechanisms, and personal circumstances were influenced differently across various populations.

Like the general population, individuals with ASD also exhibited diverse responses to these challenges, further emphasizing the variability in the pandemic’s effects on both groups. Within the reviewed studies, the impact of the pandemic on social interactions was similarly ambiguous. For instance, 47% of individuals with ASD reported increased discrimination and isolation, while 21% experienced relief from social pressure [60]. Many young people with ASD benefited from lockdowns, including reduced sensory overload and the removal of the need to mask autistic traits [82]. It is important to note that, despite the centrality of sensory sensitivities and masking in the lived experience of autistic individuals, only one study addressed these topics, highlighting a significant gap in the current literature.

On the other hand, social restrictions and limited, inadequate support contributed to increased stress and anxiety among individuals with ASD, highlighting the need for specialized psychological interventions [83]. Similarly, while reduced social contact was linked to heightened anxiety and depression in some individuals with ASD and their caregivers, these effects were not universally observed. This underscores the importance of providing emotional support during prolonged crises, though the impact of such crises may vary depending on individual circumstances [84].

Contrary to the common belief that the pandemic universally worsened ASD symptoms, a systematic review revealed that behavioral traits such as irritability and social withdrawal did not significantly deteriorate in all individuals with ASD [59]. These findings align with observations that nature served as a refuge for many adults with ASD, helping reduce stress and improve emotional well-being [85]. The opportunity to reconnect with nature as a coping mechanism emphasizes the importance of developing support strategies that integrate outdoor environments and safe spaces for relaxation.

Furthermore, the pandemic shifted the perception of digital platforms as supportive tools. Virtual reality-based telerehabilitation improved motor coordination and manual dexterity in individuals with ASD while increasing receptivity from parents and children towards these interventions [86]. Despite challenges in accessing in-person services, these digital solutions proved to be a viable alternative for rehabilitation and motor skill development. However, barriers to healthcare access did not significantly decrease during the pandemic; instead, they evolved, raising concerns about equitable access to technological tools for all individuals with ASD [84].

Several studies have also examined how autistic traits influenced emotional responses to the pandemic. Brosnan and Gavin [87] found that autistic traits significantly mediated the relationship between the impact of the lockdown and the risk of hikikomori, especially in individuals who had been in lockdown for six months or more. Higher levels of autistic traits were associated with lower psychological well-being, both directly and indirectly. Similarly, Shakeshaft et al. [88] reported increased anxiety and worsened emotional well-being in individuals with elevated autistic traits during the pandemic. These findings highlight the importance of considering autistic traits when examining the psychological impact of prolonged lockdowns on young adults with autism.

The authors of this SR recognize that conceptualizations of autism can vary considerably depending on the theoretical frameworks from which the condition is approached. Such differences may have a direct impact on key methodological decisions, including the criteria used to determine participant inclusion in research studies. In this SR, the authors adopted a broad and inclusive perspective of the autism spectrum, acknowledging the heterogeneity of individual profiles and levels of support required, including cases involving intellectual disability (ID). This decision reflects their intention to represent the clinical and functional diversity inherent to the spectrum [10,89] and to more comprehensively address the realities and needs of the autistic population.

Nonetheless, the authors are cognizant that alternative approaches advocate for more narrowly defined research samples, based on the concern that the inclusion of individuals with ID may obscure the identification of autism-specific characteristics, particularly in studies focused on cognitive functioning or social interaction within neurotypical contexts [90]. These contrasting positions reflect legitimate and ongoing debates within the field and highlight the importance of explicitly articulating the conceptual frameworks that inform empirical investigations. The authors contend that this plurality of perspectives contributes to the enrichment of the field and promotes a necessary dialogue aimed at advancing a more comprehensive and nuanced understanding of autism.

Despite the valuable insights, a key limitation of this review is the scarcity of studies included, which is related to other limitations we will address below. Another significant limitation was the age range of young adults and the changes made in the decision-making process regarding this range.

A further limitation is the insufficient focus on young adults with ASD as a distinct population. Most available research combine broad age groups and employ varying methodologies, complicating direct comparisons and limiting the generalizability of findings. Some studies include individuals up to 30, while others restrict the sample to younger participants. This inconsistency in defining “young adults” made establishing a clear and universally applicable age boundary challenging. To address this, we removed the predefined age restriction of 18–25 years in our initial protocol. This allowed us to include and analyze studies that considered young adults within a broader age range capturing relevant data. This decision was also guided by the concept of “emerging adulthood” proposed by Jeffrey Arnett [91]. This framework recognizes that key developmental processes—such as identity formation, career development, and relationship building—extend into the late twenties and early thirties. This is especially relevant for individuals with ASD, as the transition to independent adulthood can occur at different times depending on individual needs and support [92,93,94]. Cultural and socioeconomic factors also influence the timing of transitions to adulthood, further highlighting the need for a broader age range. This adjustment allows for a more accurate representation of the existing literature and provides a more comprehensive view of the diverse experiences of young adults with ASD.

Moreover, during an unprecedented global crisis, the research had limitations and methodological differences. The pandemic influenced research efforts, affecting the quantity and quality of available studies. Due to difficulties recruiting participants, many studies relied on pre-existing cohorts or qualitative methods with small sample sizes, limiting their generalizability. The urgency of conducting research during a rapidly evolving crisis also led to methodological inconsistencies. This limitation has significant implications. The small body of evidence and heterogeneity in study designs make the results more susceptible to individual study biases, restricting the generalizability of conclusions. Therefore, findings should be interpreted with caution.

Another limitation of this review was the heterogeneity in methodologies and outcome measures across the included studies. Differences in study design, sample sizes, participant characteristics, and types of outcomes assessed complicated the extraction of definitive conclusions. Additionally, a further challenge was the high prevalence of co-occurring health and mental health conditions (83%) within the autistic young adult population [95], making it challenging to identify studies focused solely on young adults with autism without comorbidities. Our SR highlights these gaps, underscoring the need for more targeted research focusing on age-specific experiences, consistent methodologies, and clinical profiles. While the search strategy was systematic, it may have inadvertently excluded relevant literature due to limitations in database selection, search terms, or publication restrictions. Studies were excluded for not meeting the established age criteria, such as those focusing solely on children, including adults without age disaggregation, or lacking age-related data. Additionally, studies involving populations not directly relevant to the review, such as parents, clinicians, or individuals with other diagnoses, were excluded.

Another limitation was the difficulty in finding studies that explored how the effects of the pandemic evolved in this population, including both immediate and longer-term impacts. Additionally, the long-term effects of COVID-19 on individuals with ASD remain largely unknown, highlighting the need for further research to understand its sustained impact on mental health, social adaptation, and overall well-being.

The COVID-19 pandemic highlighted significant gaps in research and support for young adults with ASD. Future research should focus on the long-term effects of the pandemic on mental health, social adaptation, and well-being. Standardizing age ranges and methodologies will improve comparability across studies. Additionally, tailored support for caregivers is crucial, as their burden increased during the pandemic. Digital interventions, such as virtual health services, should be further explored for their long-term effectiveness. Employment opportunities for individuals with ASD need to be more flexible, and interventions should be personalized to address the varied emotional responses to social isolation. Lastly, international guidelines should be strengthened to support individuals with ASD during global crises.

## 5. Conclusions

In conclusion, the COVID-19 pandemic, declared a global health emergency, prompted swift and widespread public health measures such as isolation, quarantine, and social distancing to limit the virus’s transmission. These measures had a profound impact on young adults with ASD, disrupting their daily routines and exacerbating challenges such as increased dependence on caregivers, disruptions in routine, and limited access to essential services. However, some individuals with ASD displayed remarkable adaptability during this period, with improvements in autonomy resulting from the increased use of remote work and virtual healthcare services. Digital interventions and structured support systems played a crucial role in alleviating some of the negative effects of these disruptions.

Despite considerable methodological heterogeneity among the reviewed studies (designs, outcome measures, and sample characteristics), several important patterns emerged, including heightened emotional stress, increased social difficulties, and the critical importance of providing tailored support. These findings underscore the need for inclusive policies and long-term strategies that address the specific needs of young adults with ASD, especially during public health emergencies. Future research should focus on exploring the long-term impacts of the pandemic on this population and developing more effective interventions to support their well-being in future crises, ensuring a more resilient response to similar challenges.

## Figures and Tables

**Table 1:** General data extraction from eligible articles.

Authors	Aim of the Study	Country of Sample	Study Design	Age (M/SD)	Gender Distribution (Female/Male/Others)	Groups Comparison	COVID-19 Phase
Brondino et al. [59]	This study aimed to evaluate the impact of COVID-19 restrictions on challenging behaviors in individuals with severe ASD.	Italy	Longitudinal	22.72 ± 4.75	27.8%/72.2%	Autistics before vs. Autistic during the COVID-19 restrictions	First stage (between 19 February 2020 and 4 March 2020).
Davidson and Pfeiffer [60]	This study explores community participation barriers and facilitators for autistic individuals during COVID-19, using Photovoice methodology.	USA	Cross-sectional	20.5 ± 1.44	18%/76%/6% others	Autistic individuals with vs. without co-occurring mental health conditions during COVID-19.	Early COVID-19 (summer of 2020)
Mosquera et al. [61]	This study explored the lived experiences of autistic adults regarding social expectations before and during the first COVID-19 lockdown in Spain.	Spain	Longitudinal	30.2 ± 5.06	20%/60%/20% non binary	Autistic before vs. Autistic during the first COVID-19 lockdown in Spain.	First COVID-19 lockdown
Pfeiffer et al. [62]	This study examined the impact of COVID-19 on community mobility and participation for young adults with ASD.	USA	Longitudinal	23.5 ± 2.4.	33.33%/66.67%	Autistic Before vs. Autistic after the COVID-19 pandemic.	Beginning of the COVID-19 lockdown (between March 20th and April 2nd/between April 20th and May 3rd).
Schnitzler et al. [63]	This study investigated how partial face covering, such as masks, affects emotion recognition in individuals with ASD.	Germany	Cross-sectional	28.08 ± 9.09	19.44%/80.56%	ASD vs. control.	From August to December 2021, during the mandated outdoor mask-wearing period.
Taylor et al. [64]	This study examined whether employment changes due to COVID-19 predicted increased depressive symptoms in young adults with ASD.	USA	Longitudinal	26.5 ± 4.91	46.9/47.6%/5.6% others	ADS with employment changes during COVID-19 vs. ADS no employment changes.	Initial reopening phase (May 2020).
Tovin & Núñez-Gaunaurd [65]	This study examined the feasibility and acceptability of the Physical Activity Connections program via Telehealth, implemented during the COVID-19 pandemic lockdown as an alternative to in-person programming for autistic adults.	USA	Longitudinal	25.83 ± 3.91	38.8/61.1%	ASD physical activity intervention vs. ASD without physical activity intervention	COVID-19 quarantine lockdown
Valenti et al. [66]	This study aimed to evaluate the adaptive behavior of young adults with ASD.	Italy	Longitudinal	21.95 ± 5.78	31.82%/68.18%	ASD with no access to in-person care (only telehealth) vs. ASD with access to in-person care	During the first COVID-19 lockdown (T0), after 6 months (T1), and after 1 years (T2).

**Table 2:** Summary of key findings from the reviewed studies.

Authors	Domains Covered	Main Findings
Brondino et al. [59]	Behavioral and Cognitive	No significant changes in behaviors after COVID-19 restrictions.
Davidson and Pfeiffer [60]	Daily Life and Accessibility	Transportation was identified as a barrier 32% of the time and a facilitator 0%.
	Social and Emotional Impact	COVID-19 was identified as a barrier 47% of the time and facilitator 21% of the time.
Mosquera et al. [61]	Daily Life and Accessibility	Participants noted several impacts on their daily lives. Anxiety and insecurity grew due to public insults, but some felt less pressure to mask autistic traits. The extra time for personal interests and family was valued, and applause for healthcare workers was seen as a symbol of unity. Remote work provided new inclusion opportunities, while shortages in specialized services limited access to care and support.
Pfeiffer et al. [62]	Daily Life and Accessibility	Significant reduction in daily activities, travel, and transportation; mobility remained lower than pre-pandemic levels (GPS data).
Schnitzler et al. [63]	Social and Emotional Impact	ASD group had more difficulties recognizing emotions (fear, pride, embarrassment and anger) than the non-ASD group, especially with masks/sunglasses.
	Behavioral and Cognitive Outcomes	There were no significant differences between the ASD and non-ASD groups in emotion recognition or spatial distance, as both groups maintained similar interpersonal distances.
Taylor et al. [64]	Daily Life and Accessibility	A majority of autistic individuals reported employment disruptions during the pandemic, while 30% experienced positive changes. Negative perceptions of these changes were associated with increased depressive symptoms.
Tovin & Núñez-Gaunaurd [65]	Social and Emotional Impact	Employment and Mental Health: Job loss linked to increased depressive symptoms (+3.76 points) in adults with ASD.
	Social and Emotional Impact	Physical Activity via Telehealth: 80% attendance; <20% dropout; high satisfaction and goal achievement; technology boosted engagement.
Valenti et al. [66]	Daily Life and Accessibility	Autonomy declined, especially in hygiene, dressing, and eating, with younger individuals being more affected. Disruptions in routines also led to a decrease in domestic skills and greater reliance on parents.
	Social and Emotional Impact	Interpersonal Relationships: Significant reduction between T0-T2 and T1-T2; isolation and social routine disruption likely increased stress and anxiety.

## Data Availability

Not applicable.

## Supplementary Materials

The following supporting information can be downloaded at: https://www.mdpi.com/article/10.3390/healthcare13111216/s1. Methodological Quality of the Studies and Risk of Bias (Supplementary S1).The results of the database search and any materials used in this SR are available from the authors upon request.
