# Supplementary material for: The Impact of the COVID-19 Pandemic on Young Adults with Autism Spectrum Disorder: A Systematic Review
Source: Healthcare (Basel). 2025 May 22;13(11):1216. doi: 10.3390/healthcare13111216 (PMC12155291; doi:10.3390/healthcare13111216)
Supplement: Supplementary file 1 [file healthcare-13-01216-s001.zip › healthcare-3592962-supplementary.pdf]

Appendix 1: Critical Appraisal Skills Programme (CASP) for studies revised according to specific checklists. *Source: https://casp-uk.net/casp-tools-checklists/*

COHORT STUDY CHECKLIST

**Study**	**Researcher**	**1**	**2**	**3**	**4**	**5(a)**	**5(b)**	**6(a)**	**6(b)**	**7**	**8**	**9**	**10**	**11**	**12**	**QUALITY**	**Final Quality Assessment**
**Brondino et al.** [61]	ALM	Y	Y	Y	CT	Y	CT	Y	Y	CT	N	Y	CT	N	N	Moderate	Moderate
	APP-U	Y	Y	Y	Y	CT	CT	Y	N	Y	Y	CT	CT	N	N	Moderate	
**Pfeiffer et al.** [64]	ALM	Y	Y	Y	CT	Y	CT	Y	Y	CT	N	Y	CT	N	N	Moderate	Moderate
	APP-U	Y	CT	Y	CT	Y	N	CT	N	Y	CT	Y	CT	Y	CT	Low/Moderate	
**Taylor et al.** [66]	ALM	Y	Y	Y	Y	Y	CT	Y	CT	Y	Y	Y	N	Y	Y	High	Moderate/High
	APP-U	Y	Y	Y	Y	CT	CT	Y	N	Y	Y	Y	N	Y	CT	Moderate/High	
**Tovin & Núñez-Gaunaurd** [67]	ALM	Y	Y	CT	Y	Y	CT	Y	Y	Y	Y	Y	CT	Y	CT	High	High
	APP-U	Y	Y	Y	Y	Y	CT	Y	CT	Y	Y	Y	N	Y	Y	High	
**Valenti et al.** [68]	ALM	Y	Y	Y	Y	CT	CT	Y	N	Y	Y	Y	N	Y	CT	Moderate/High	High
	APP-U	Y	Y	CT	Y	Y	CT	Y	Y	Y	Y	Y	CT	Y	CT	High	

CROSS-SECTIONAL STUDIES CHECKLIST

Study	Researcher	1	2	3	4	5	6	7	8	9	10	11	QUALITY	Final Quality Assessment
**Schnitzler et al.** [65]	ALM	Y	Y	Y	Y	Y	Y	Y	Y	Y	Y	Y	High	High
	APP-U	Y	Y	Y	Y	Y	Y	Y	Y	Y	Y	Y	High	

QUALITATIVE RESEARCH CHECKLIST

Study	Researcher	1	2	3	4	5	6	7	8	9	10	QUALITY	Final Quality Assessment
**Davidson & Pfeiffer** [62]	ALM	Y	Y	Y	CT	CT	Y	Y	Y	Y	CT	Moderate	Moderate/High
	APP-U	Y	Y	Y	CT	Y	Y	Y	Y	Y	CT	Moderate/High	
**Mosquera et al.,** [63]	ALM	CT	CT	Y	Y	Y	Y	Y	Y	Y	Y	Moderate	Moderate
	APP-U	Y	Y	Y	CT	CT	Y	Y	Y	Y	CT	Moderate	
